# Cross-Cultural Validity of a Dietary Questionnaire for Studying Dental Caries in Japanese Children

**DOI:** 10.3390/healthcare11071036

**Published:** 2023-04-04

**Authors:** Yukie Nakai, Yukako Mori-Suzuki

**Affiliations:** 1 Department of Dental Hygiene, University of Shizuoka Junior College, Shizuoka 422-8021, Japan; 2 Suzuki Dental Clinic, Hiroshima 732-0822, Japan

**Keywords:** cariogenic diet, dietary sugars, non-communicable diseases, oral health, factor analysis

## Abstract

This study examines the cross-cultural validity of the Japanese version of the Food Frequency Questionnaire (FFQ), designed for studies on dental caries to assess dietary intake in Japanese children. Parent-reported dietary data were collected (274 children, 3–6 years old) using the 38-item FFQ, whose reliability and validity have been demonstrated in adults. Factor analysis was used to determine dimensionality. Dietary cariogenicity scores were compared with the levels of plaque mutans streptococci, and the decayed, missing, and filled teeth (dmft) index to evaluate the criterion validity using Pearson’s correlation coefficient (*r*) and the Kruskal–Wallis test. The FFQ showed good criterion validity, assessed through its relationship with the dmft index (*r* = 0.119; *p* = 0.05) and Dentocult SM score (*r* = 0.124; *p* = 0.04). Factor analysis revealed six questionnaire subscales. Internal consistency was from low to acceptable (Cronbach’s alpha = 0.64 for the total scale, 0.39–0.53 for each subscale). Children with a higher SM score were more likely to have higher dietary cariogenicity scores (*p* = 0.01; Kruskal–Wallis test). These results confirm the validity of the Japanese version of the FFQ for children, which can be used to track dietary structure dynamics regarding cariogenicity from childhood to adulthood.

## 1. Introduction

Dental caries is currently considered a non-communicable disease, like diabetes, heart disease, stroke, kidney disease, mental and neurological disorders, chronic respiratory diseases, and cancers [1]. Although the etiologies of these diseases are different, they share common risk factors, such as excess sugar consumption, alcohol and tobacco use, malnutrition, and low physical activity. Dental caries has a multifactorial etiology, including susceptible teeth due to enamel defects such as hypoplasia, oral colonization with cariogenic bacteria (especially mutans streptococci (MS)), and sugar [2]. Previous studies also suggested oral hygiene, breastfeeding, and socioeconomic status as caries risk factors [3] and possible causing factors of hypoplasia [4]. The oral microbiome in health and disease is considered an ecological factor that could shift from symbiosis to dysbiosis under various environmental changes that act as drivers [5]. Environmental changes due to acid production from fermenting dietary carbohydrates, especially sugars, work with acidogenic and aciduric bacteria, such as MS and lactobacilli, disrupting the symbiotic balance of dental biofilms. This leads to dysbiosis, which favors dental caries initiation and progression. Despite changes in caries conceptualization, dietary sugar consumption remains a modifiable major etiological factor in the development of dental caries. The Global Burden of Disease Study reported that untreated caries in permanent teeth was the most prevalent in 291 diseases and injuries evaluated for their global burden. The study also reported that severe periodontitis and untreated caries in deciduous teeth were the 6th and 10th most prevalent conditions, respectively [6]. Approximately 3.9 billion individuals globally are affected by oral diseases [6]. According to a statement by the FDI World Dental Federation and International Association for Dental Research, sugar is the primary factor in dental caries development, which is the most widespread and preventable non-communicable disease [1]. As habitual sugar consumption in childhood and adolescence is a significant risk factor for dental caries, diabetes, obesity, and cardiovascular diseases, the public health and clinical perspectives of the concept of the common risk factor approach need to be addressed [7].

A Finnish longitudinal study that examined individuals at 3, 6, 12, and 16 years old demonstrated that high sugar consumption at 3 years was associated with a high salivary MS content and a high caries index in later years [8]. A Brazilian longitudinal study that examined individuals at ages 6, 12, and 18 revealed similar findings: high and upward sugar consumers had higher dental caries prevalence and mean DMFT index in later years [9]. These studies indicated that, when the habit of high sugar consumption was formed at a younger age or during the early childhood, it continued throughout the follow-up period, suggesting that major improvements are less likely to occur later in life. As the early life is a critical period for developing sensory perception and food preferences, dietary habits during this period serve as the foundation for the development of food preference for the entire life [10]. Controlling dietary sugar in younger children may have beneficial long-term effects on oral health. Taking a life course perspective can bring a better understanding to the model of disease development and aid in finding cost-effective preventive strategies for diet-related chronic diseases [11]. Investigating changes in dietary risk over time requires instruments that could be used in both child and adult subjects to obtain measurements over time. However, besides some food frequency questionnaires (FFQ) that are valid for either children or adults, such instruments remain lacking [12,13,14,15,16,17].

Although the diet is a central component of oral and general health, and its importance in childhood cannot be overemphasized, information on the relationship between diet and childhood caries is sparse in Japan [18]. Identifying children at high caries risk could be the first step to sharing information that could improve oral and general health. A possible reason for the sparsity of this information in Japan is that assessing dietary habits in children is challenging. Moreover, there are limited valid assessment tools for Japanese children, and methodological problems because of the limited cognitive ability of children. The Japanese version of the FFQ, which was designed specifically for dental caries studies [12], exhibited good reliability and validity in adults [13]. The feasibility of the FFQ in children is unclear. Children under 8 years cannot accurately report their food consumption; thus, acquiring dietary information from such young children depends on assistance from their parents or other guardians [19]. In a previous study, the correlation coefficient between levels of MS in saliva, a caries risk indicator, and the dietary cariogenicity score calculated from the FFQ was used to assess the criterion validity of the FFQ [13]. The MS level in the plaque on the tooth surface is considered more suitable as an ecological driver than the MS level in saliva on the basis of the ecological plaque hypothesis [5]. Further, the caries outcome should be used as the gold standard. Therefore, in the present study, we examine plaque MS levels and caries occurrence to confirm the criterion validity of the FFQ.

The aim of this study is to confirm the cross-cultural validity of the Japanese version of the FFQ, designed for studies on dental caries to assess the dietary intake of Japanese children in order to plan investigations from childhood to adulthood, with the secondary long-term aim of providing preventive information on diet-related chronic diseases as a common risk factor. The specific objectives are to: (1) investigate the construct validity of the questionnaire through factor analysis and examine its internal consistency reliability, and (2) assess its criterion validity in terms of the relationship between caries occurrence and plaque MS levels.

## 2. Materials and Methods

### 2.1. Participants

The participants of this cross-sectional observational study were consecutive pediatric patients and their parents, seen between December 2016 and October 2018 in a private practice dental office in Kurashiki, Okayama, Japan. Kurashiki is the second largest city in the Okayama prefecture, with 48,400 inhabitants, with a low fluoride concentration (approximately 0.11 ppm) in the drinking water supply. The dental office was located inside a large shopping mall in the central city, with easy access from the nearest railway station or by vehicle. 

All procedures were performed according to the stipulations of the Declaration of Helsinki. The institutional review board of the University of Shizuoka approved the study (approval number: 29–29). The parents provided their written informed consent on behalf of their children. Eligible child participants were 3–6 years old (with primary dentition), were healthy, and their parents were able to communicate in Japanese. Participants were excluded if any permanent teeth had erupted or if their parents reported antibiotic use during the prior month. 

The sample size was estimated on the basis of the assumption that the correlation coefficient between MS level and the dietary cariogenicity score was 0.20 [13]. If the power was set to 90%, and the significance level was <0.05, the minimal sample size was 259. The subjects were 274 pediatric patients (mean 4.4 ± 1.1 years).

### 2.2. Measures and Procedures

The parents were invited to answer the questionnaire, which comprised (1) child characteristics (age, sex, birth weight, and type of health insurance), (2) toothbrushing habits, and (3) the food frequency questionnaire (FFQ). The parent-reported child dietary data were collected in the waiting room during a dental visit, and questionnaires were administered by trained dental hygienists blinded to the oral examination results. Oral examination and plaque sampling were performed on the day of the dietary assessment.

### 2.3. Food Frequency Questionnaire (FFQ)

The parents were asked to complete the Japanese version of FFQ for their children. The English version of the questionnaire had been translated into Japanese and then back into English. Its reliability and validity were examined for adults (see details in the study by Shinga-Ishihara et al. [13]). 

The FFQ includes 38 items on food habits requiring the respondents (parents) to recall how often, on average, their children had consumed a given caries-related food during the past month (frequency score: 7 points, 0–6, ranging from never to 4 or more times per day). The questionnaire was scored by multiplying the frequency score for each item by a cariogenicity rating (3 points; 0–2, from possibly to highly cariogenic) on the basis of the Palmer cariogenicity classification [20]. Next, the products were summed to create the overall score (the dietary cariogenicity score), ranging from 0 to 378.

### 2.4. Dental Caries

Oral examinations were conducted in a fully equipped dental chair by a single experienced pediatric dentist whose caries diagnostic ability had been compared with that of an expert (inter- and intraexaminer reliability; both κ > 0.8) according to the criteria of the World Health Organization (WHO) [21]. Dental caries assessments were performed using an explorer and a mouth mirror under good illumination and with radiographic assistance when necessary. According to the WHO criteria [21], caries are lesions with a detectable cavity and an undermined enamel in a pit, fissure, or smooth surface. Decayed (cavitated), missing (due to caries), and filled primary teeth were counted to record the dental caries experience (the dmft index). The children were dichotomized as caries-positive (dmft ≥ 1) or caries-free (dmft = 0) on the basis of their caries status.

### 2.5. Plaque Mutans Streptococci Levels

The levels of plaque mutans streptococci were estimated using the Dentocult^®^ SM Strip mutans kit (Oral Care Co., Tokyo, Japan) following the manufacturer’s instructions. For each child, samples were obtained from the tooth surfaces. A microbrush (Benda Microtwin; Centrix, Inc., Shelton, CT, USA) was used to collect plaque samples from the buccal cervical regions of the tooth surfaces; dental floss was also used for all approximal surfaces of the erupted teeth. These collection methods had been validated by Karjalainen et al. [22]. Plaque samples were spread onto 1 of 4 roughened sites of the site strip (Site 1, upper cervical; Site 2, lower cervical; Site 3, upper approximal; Site 4, lower approximal). The inoculated site strip was inserted into the culturing vial in the test kit. After incubation at 37 °C for 48 h, a researcher assessed bacterial growth on the strips with a density chart whose intraexaminer reliability was κ > 0.8. SM scores 0–3 were used, corresponding to <10^4^, 10^4^–10^5^, 10^5^–10^6^, and 10^6^ > CFU/mL of saliva, respectively. The maximal score of the four sites was given as the representative SM score.

### 2.6. Data Analysis

The distribution of the 38 items was examined to assess the amount of missing data. Out of the 275 enrolled participants, 274 questionnaires were completed and used in the study. The internal consistency reliability was assessed using Cronbach’s alpha. Factor analysis was used to examine dimensionality. Principal axis factoring with Varimax rotation was employed. Kaiser’s criterion (eigenvalues > 1.0) and a visual scree plot were utilized to determine the number of components to retain. Internal consistency was assessed for the full scale and subscales developed from the factor analysis. Criterion validity was assessed by examining the relationships between the dietary cariogenicity and dmft scores, and between the dietary cariogenicity and Dentocult SM scores using Student’s *t*-test, Pearson’s correlation coefficient (*r*), and the Kruskal–Wallis test. Statistical analyses were performed using IBM SPSS version 23 software (IBM Corp., Armonk, NY, USA).

## 3. Results

In total, 274 parents completed the questionnaire (99.6%). The mean dmft of the children was 2.3 ± 3.8 (± standard deviation (SD); range, 0–20), and the prevalence of dental caries was 39.8%. Table 1 shows the children’s characteristics and compares each item between children with and without dental caries. Children with dental caries were significantly more likely to have high plaque SM scores than those without (*p* < 0.001, χ^2^ = 35.158; chi-squared test). Toothbrushing habits, such as toothbrushing frequency and the presence of parental supervision, were not significantly associated with dental caries. No significant difference was found in the type of health insurance between groups, and there was no recipient of the public assistance system. Considering discriminant validity, a significant difference was observed in the dietary cariogenicity score calculated from the FFQ between children with and without dental caries (*t* = −2.488, *p* = 0.013). The distribution of the frequency scores for each food item is given in Table 2. 

### 3.1. Construct Validity and Internal Consistency Reliability

The appropriateness of the data for factor analysis was verified using the Kaiser–Meyer–Olkin measure (KMO = 0.625), which was above the acceptable limit of 0.5. Bartlett’s test of sphericity (χ^2^ (703) = 1513.811, *p* < 0.001) indicated that the correlations between items were sufficiently large for analysis. The factor analysis of the patients’ samples using Kaiser’s criterion revealed that 15 factors had eigenvalues >1, which accounted for 61.8% of the variance in the total scores. The scree plot confirmed the retention of the first six factors. Table 3 shows factor loadings after rotation and the six factors. Cronbach’s alpha for the total scale was 0.64. The alphas for each subscale were 0.52, 0.48, 0.54, 0.48, 0.39, and 0.53. The mean cariogenicity subscores for each subscale were 5.8 (SD = 3.1, range 0–18), 5.3 (4.1, 0–22), 3.6 (2.9, 0–14), 3.4 (2.5, 0–12), 0.8 (2.0, 0–16) and 0.2 (1.1, 0–14).

### 3.2. Criterion Validity

**Figure 1:**
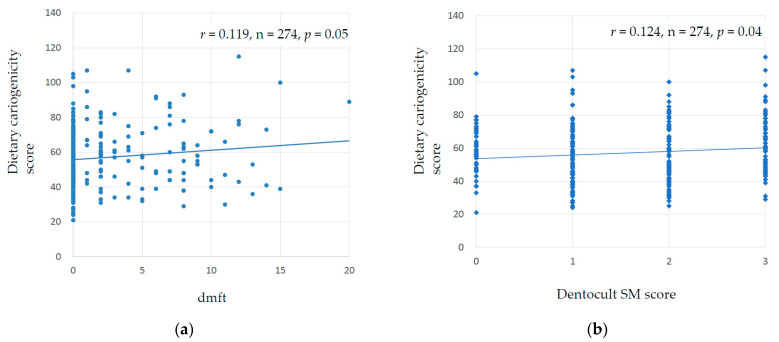
Scatter plots of the relationship between (**a**) dietary cariogenicity score and dmft index, and (**b**) dietary cariogenicity and Dentocult SM scores.

The mean dietary cariogenicity score derived from the Japanese version of the FFQ was 57.0 ± 17.3 (range, 21–115). Children with dental caries had significantly higher dietary cariogenicity scores than those without (60.1 ± 18.7 vs. 54.9 ± 16.0; Student’s *t*-test, *p* = 0.013) (Table 1). Figure 1a shows a scatter plot of the relationship between the dietary cariogenicity score and dmft index, and Figure 1b shows the relationship between the dietary cariogenicity and Dentocult SM scores. The dietary cariogenicity score was positively correlated with measures of caries experience (*r* = 0.119, *p* = 0.05) and plaque MS levels (*r* = 0.124, *p* = 0.04). Table 4 shows the mean dietary cariogenicity score for each category of Dentocult SM score. Children with higher SM scores were more likely to have higher dietary cariogenicity scores (*p* = 0.01; Kruskal–Wallis test).

## 4. Discussion

This study is the first to investigate the feasibility of a parent-reported version of the FFQ for studying dental caries in Japanese children. The FFQ, which is reliable and valid in adults, was also confirmed as valid in children. Criterion validity was confirmed by examining the associations between FFQ cariogenicity score and plaque MS levels, and between the FFQ cariogenicity score and caries outcome. Factor analysis suggested six factors: solid sugars, solid and slowly dissolving sugars, sticky sugars, semisolid and sticky sugars, slowly dissolving sugars, and starchy and slowly dissolving sugars. A previous study in an adult sample showed four factors, one of which was termed liquid and semisolid sugars, including cold drinks, not-diet soda, and ice cream/sorbet [13], which did not appear in the child sample. There were 23 food items that showed at least a 10% difference in one of the frequency scores between the results of this and of a previous study on adults [13]. The structure of food consumption may differ between adults and children due to generational variations in consumption-related behaviors and dietary preferences. In a study in New Zealand, Amezdroz et al. reported that children’s diets became increasingly cariogenic with age as a wider variety of foods and drinks were introduced [14]. However, this study shows a contrary result: the mean dietary cariogenicity score in the children was higher than that in the adults of the previous study [13]. We speculate that the cariogenicity of the diets increases from childhood until a certain age and then decreases according to those findings. This scale provides a measure to track the dynamics of the dietary structure regarding cariogenicity from early childhood to adulthood.

Cronbach’s alpha for the total scale was similar to that in the previous study [13]. Cronbach’s alpha of the six subscales showed relatively low values in the range of 0.39–0.54 due to the few food items on each subscale (Factors I and II, 3 items; the other factors, 2 items), since the alpha value depends on the number of items on the scale and the correlation between them. Therefore, the alpha value increases as items on the scale increase. The discriminatory validity of the FFQ to distinguish between children with and without dental caries on the basis of their dietary cariogenicity score was established in this study. It confirmed that higher scores indicated a higher caries risk of dietary factors.

This study has some limitations. First, the FFQ assesses the consumption frequency of certain foods and beverages, and not their timing (at meal time or between meals) or simultaneous consumption. Second, the dietary measures do not consider xylitol. Xylitol-containing products, which have become more commonly available in Japan in recent decades, exert anti-caries effects. Further, this scale may underestimate the contribution of timing, food combinations, and other popularly consumed foods and beverages to the score. Third, parental recall bias may have affected the accuracy of the data and obtained results. Fourth, the possibility that age is a confounding factor in caries occurrence cannot be ruled out, since this study found a significant difference in age between those with and without dental caries. The most likely reason why children with caries are significantly older than those without could be the increased number of erupted teeth and the longer exposure of erupted-tooth surfaces to the oral environment. Since primary dentition was set as an inclusion criterion in this study, 6-year-old children without permanent teeth were included. In this study, the number of erupted teeth was consistent across ages, regardless of the presence or absence of caries. We assumed that differences in physiological age in relation to development such as tooth eruption were the least despite the chronological age differences.

Ethnicity and socioeconomic status (SES) are associated with dental caries [23,24,25]. We did not collect information regarding race, education, and annual income, since the questionnaire was not anonymous, and we aimed to maximize the response rate. However, Japanese was the only race included in this study. Poor levels of SES (as indicated by the public assistance system that supports households whose income is below the standard set by the government) were not included, and there was no significant difference in insurance types. This indicates that the participants were culturally and economically almost homogeneous. However, there remains the possibility that SES has contributed to dental caries in this study population. Stein et al. [26] reported that SES and dietary patterns independently affected tooth loss and dental caries among adults in the United States (US). The authors speculated that sugary foods and drinks were highly consumed by rich and poor US citizens regardless of their SES [26]. Future work needs to examine the link between SES and diet in the Japanese population. 

The study’s strengths include using indices that are more fitted for caries etiological models such as plaque MS and caries experience to assess validity compared with previous studies. Additionally, possible confounding factors such as toothbrushing habits could be de-emphasized since they were not significantly associated with caries outcome in the study sample. Only one questionnaire was not completed in our study (0.4%; 1/275), indicating high internal validity. Quantifying the degree of dental caries risk using the dietary cariogenicity score derived from the FFQ is easy. Other medical and health professionals could easily apply the scale to identify gradual changes in a person’s diet between specific time points in order to enlighten them on common risk factors for dental caries development. The scale could be used as a tool to improve the understanding of the relationship of the diet to oral-health and other conditions, such as obesity and overweight status [27,28,29].

The parent-reported version of the FFQ exhibited reliability in internal consistency, and good construct and criterion validity in a population of Japanese children. These findings agree with those of other food frequency questionnaires [30], and suggest the applicability of the Japanese version of the instrument in dental caries research. Further confirmation is necessary to generalize the findings to other age ranges of the population. Future studies should consider the potential protective factors of some dietary components.

## 5. Conclusions

The Japanese version of the FFQ is a reliable and valid instrument for assessing dietary intake in relation to dental-caries risk and occurrence in children. The questionnaire could be applied to both Japanese and Western cultures. Further confirmation is needed to verify the usefulness of the questionnaire.

## Figures and Tables

**Table 1:** Participant characteristics and comparisons between children with and without caries.

	Total (*n* = 274)		With Caries (*n* = 109; 39.8%)		Without Caries (*n* = 165; 60.2%)		
		Column		Row		Row	
	*n*	%	*n*	%	*n*	%	*p*-Value
Sex							
Female	137	50.0	56	40.9	81	59.1	0.805 ^a^
Male	137	50.0	53	38.7	84	61.3	
Plaque SM scores							
0	40	14.6	12	30.0	28	70.0	<0.001 ^b^
1	108	39.4	26	24.1	82	75.9	
2	67	24.5	30	44.8	37	55.2	
3	59	21.5	41	69.5	18	30.5	
Toothbrushing/day							
0	0	0.0	0	0.0	0	0.0	0.119 ^b^
1	56	20.4	19	33.9	37	66.1	
2	115	42.0	49	42.6	66	57.4	
3	100	36.5	38	38.0	62	62.0	
≥4	3	1.1	3	100.0	0	0.0	
Parental supervision for toothbrushing							
Always	240	87.6	92	38.3	148	61.7	0.197 ^a^
Sometimes	34	12.4	17	50.0	17	50.0	
Never	0	0.0	0	0.0	0	0.0	
Type of health insurance							
Employee	249	90.9	95	87.2	154	93.3	0.09 ^a^
National	25	9.1	14	12.8	11	6.7	
Public assistance	0	0.0	0	0.0	0	0.0	
	Mean	SD	Mean	SD	Mean	SD	*p*-value
Age (years)	4.4	1.1	4.9	1.0	4.2	1.0	<0.001 ^c^
Birth weight (g)	3017.4	407.9	3035.9	359.9	3005.1	437.3	0.542 ^c^
Dietary cariogenicity score	57.0	17.3	60.1	18.7	54.9	16.0	0.013 ^c^

SM scores reflect the levels of mutans streptococci (MS) obtained with the Dentocult SM Strip mutans test. SD, standard deviation; ^a^ *p*-values from Fisher’s exact test; ^b^ *p*-values from chi-squared test; ^c^ *p*-values from Student’s *t*-test.

**Table 2:** Distribution of the frequency scores for each food item (*n* = 274).

	Distribution Percentage of Frequency Scores						
Food Item	0 (Never)	1 (Rarely)	2 (1/wk)	3 (2–3/wk)	4 (1/dy)	5 (2–3/dy)	6 (4+/dy)
Solid sugars							
Cookies	20.8	52.6	15.3	10.6	0.7	0	0
Donuts or muffins	25.2	60.6	11.3	2.9	0	0	0
Cakes or pies	27.0	67.9	3.6	1.5	0	0	0
Solid and slowly dissolving sugars							
Hard candy	29.2	37.2	12.8	16.8	2.9	1.1	0
Chips	35.8	46.0	13.1	5.1	0	0	0
Sticky candy (caramel)	73.0	16.4	4.7	4.7	0.7	0.4	0
Sticky sugars							
Jam, jelly, or syrup	55.5	21.5	5.8	10.6	5.8	0	0.7
Bread filled with bean or fruit jam	59.5	29.6	5.8	4.7	0	0	0.4
Semisolid and sticky sugars							
Plain yogurt	73.0	12.0	4.0	6.9	4.0	0	0
Banana	16.8	35.8	19.7	20.1	6.6	0.7	0.4
Slowly dissolving sugars							
Breath mints	96.4	2.9	0	0	0.7	0	0
Gum (not sugar-free)	79.2	13.1	4.4	2.2	0.7	0	0.4
Starchy and slowly dissolving sugars							
Plain cereal	95.6	2.9	0.7	0	0.7	0	0
Cough drops	96.4	2.9	0	0	0	0.7	0
Others							
Cheese	12.4	42.0	17.5	20.4	5.5	2.2	0
Sugared yogurt	24.5	31.8	8.4	21.2	12.4	1.5	0.4
Bread	4.0	17.2	17.9	31.8	27.4	0.4	1.5
Rice	0	0	0	1.1	8.8	83.9	6.2
Sugar added to Cereal	71.2	18.2	4.4	4.4	1.5	0.4	0
Lactic acid-containing drink	24.8	38.7	10.9	14.6	8.4	1.5	1.1
Canned fruit	72.6	23.7	1.8	1.5	0	0	0.4
Dried fruit	71.9	21.2	4.0	2.2	0.7	0	0
Milk	4.7	10.6	5.5	15.3	28.1	24.5	11.3
Fruit juice	63.9	26.6	3.6	5.5	0.4	0	0
Soda (not diet)	71.9	17.9	5.1	3.3	1.1	0.7	0
Cold drinks	16.1	40.1	12.8	22.3	6.2	2.2	0.4
Cocoa	75.9	13.5	3.6	4.4	2.6	0	0
Sugar or Honey in Coffee or Tea	82.8	8.8	1.5	3.6	2.6	0.7	0
Sugared cereal	99.6	0.4	0	0	0	0	0
Ice cream or sorbet	6.9	40.1	17.9	24.8	9.5	0.7	0
Pudding or custard	41.6	47.8	6.9	3.3	0.4	0	0
Jell-O (not sugar-free)	25.5	47.1	9.1	15.0	2.6	0.7	0
Chocolate	15.7	36.1	16.1	26.6	5.5	0	0
Popcorn	67.2	29.9	1.8	1.1	0.0	0	0
Rice cracker	21.5	43.4	17.2	16.1	1.8	0	0
Bun with bean jam filling	54.4	36.1	7.3	2.2	0	0	0
Rice cake	59.5	29.2	6.9	4.4	0	0	0
Bar of sweet jellied adzuki bean paste	90.9	9.1	0	0	0	0	0

Due to rounding errors, some totals are not 100%. The subscales appear in the background color.

**Table 3:** Factor loadings, eigenvalues, variance explained from principal axis factoring with Varimax rotation, and Cronbach’s alpha for each factor in the Japanese version of the FFQ (*n* = 274).

Food Item	Factor Loading Component					
	I ^a^	II ^b^	III ^c^	IV ^d^	V ^e^	VI ^f^
Cookies	**0.572**	−0.010	0.175	0.031	−0.035	−0.010
Donuts or muffins	**0.531**	0.239	0.044	−0.035	−0.048	0.004
Cakes or pies	**0.418**	0.052	0.022	−0.073	0.150	0.057
Hard candy	0.049	**0.662**	0.050	−0.061	0.039	0.024
Chips	0.118	**0.413**	−0.082	−0.110	0.024	−0.041
Sticky candy (caramel)	0.073	**0.405**	0.248	−0.003	0.204	0.007
Jam, jelly, or syrup	0.002	−0.027	**0.682**	−0.036	−0.019	0.103
Bread filled with bean or fruit jam	0.316	0.052	**0.556**	0.103	0.103	−0.067
Plain yogurt	0.012	−0.171	−0.009	**0.621**	−0.007	0.089
Banana	0.088	0.001	0.084	**0.404**	−0.127	0.017
Breath mints	−0.021	0.029	0.014	0.026	**0.859**	−0.024
Gum (not sugar-free)	−0.008	0.253	0.069	−0.071	**0.407**	0.006
Plain cereal	0.051	0.024	−0.025	0.129	−0.016	**0.710**
Cough drops	−0.021	−0.054	0.041	−0.053	−0.017	**0.554**
Cheese	−0.020	−0.147	−0.046	0.131	−0.082	0.074
Sugared yogurt	0.144	0.007	0.108	−0.159	−0.029	−0.061
Bread	0.144	0.069	0.237	0.019	0.057	0.082
Rice	−0.030	−0.081	0.081	0.047	−0.063	0.042
Sugar added to Cereal	0.080	0.095	−0.028	0.035	−0.106	−0.067
Lactic acid-containing drink	0.143	0.172	−0.016	−0.267	−0.012	−0.073
Canned fruit	0.000	0.073	0.144	0.047	−0.006	−0.052
Dried fruit	−0.059	−0.017	0.223	0.118	0.025	0.387
Milk	0.065	−0.048	0.086	0.111	0.035	0.007
Fruit juice	0.084	0.131	−0.040	0.025	0.011	−0.089
Soda (not diet)	−0.043	0.247	0.046	−0.039	0.304	−0.078
Cold drinks	0.330	0.365	0.095	−0.198	−0.046	−0.085
Cocoa	0.034	0.059	0.043	−0.135	0.002	−0.016
Sugar or Honey in Coffee or Tea	−0.029	−0.036	0.107	0.117	0.088	0.228
Sugared cereal	−0.023	−0.005	0.010	0.003	0.009	−0.013
Ice cream or sorbet	−0.006	0.227	0.054	−0.079	0.075	0.051
Pudding or custard	0.367	0.099	−0.080	0.016	−0.018	−0.049
Jell-O (not sugar-free)	0.079	0.111	0.032	0.024	−0.040	0.012
Chocolate	0.276	0.191	−0.050	−0.447	−0.015	0.156
Popcorn	0.080	0.370	−0.047	−0.060	0.051	−0.022
Rice cracker	0.264	0.176	0.292	0.287	0.024	0.030
Bun with bean jam filling	0.345	0.178	0.268	0.298	0.060	−0.019
Rice cake	0.175	0.074	−0.082	0.078	−0.009	−0.062
Bar of sweet jellied adzuki bean paste	0.016	0.077	0.032	0.055	0.070	0.057
Eigenvalue	1.566	1.551	1.252	1.219	1.150	1.131
Variance percentage	4.120	4.081	3.296	3.207	3.027	2.977
Chronbach’s alpha	0.52	0.48	0.54	0.48	0.39	0.53

Factor loadings over 0.400 appear in bold; ^a^ Factor I, solid sugars subscale; ^b^ Factor II, solid and slowly dissolving sugars subscale; ^c^ Factor III, sticky sugars subscale; ^d^ Factor IV, semisolid and sticky sugars subscale; ^e^ Factor V slowly dissolving sugars subscale; ^f^ Factor VI, starchy and slowly dissolving sugars subscale.

**Table 4:** Mean dietary cariogenicity scores for each Dentocult SM score category (*n* = 274).

	Dentocult SM Score			
	0	1	2	3
Mean ± SD	58.2 ± 15.5	54.0 ± 16.8	55.6 ± 17.7	63.0 ± 17.6
*n*	40	108	67	59

Kruskal–Wallis test, *p* = 0.01.

## Data Availability

The data that support the findings of this study are available from the corresponding author upon request.
